# Explaining age-related decline in theory of mind: Evidence for intact competence but compromised executive function

**DOI:** 10.1371/journal.pone.0222890

**Published:** 2019-09-20

**Authors:** Isu Cho, Adam S. Cohen

**Affiliations:** Department of Psychology and Brain and Mind Institute, Western University, London, Ontario, Canada; Nathan S Kline Institute, UNITED STATES

## Abstract

Previous studies suggest theory of mind (ToM) ability declines with age. However, prior tasks not only required ToM competence but also imposed high executive function (EF) demands, so decline in ToM ability could be caused by deterioration in ToM competence, EF, or both. It was predicted that if the elderly have intact ToM competence but compromised EF, then they should perform similarly to younger adults when using ToM tasks that lower executive demands, such as spontaneous-response tasks. Results showed that on tasks with reduced demands, older adults tracked belief to the same extent as younger adults, despite their declining EF. The findings support a model in which age-related decline in ToM ability is primarily caused by compromised EF, not ToM competence, suggesting that underlying ToM mechanisms are still intact in the elderly. We discuss implications of this work for competence-performance issues in ToM processing and the underlying sources of age-related deterioration of ToM.

## Introduction

The ability to imagine what other people are thinking, including thoughts that differ from one’s own, is known as theory of mind (ToM). Converging evidence suggests ToM ability declines with age [1]. However, given that ToM ability depends on at least ToM-specific processes (including inferring mental states from observed behavior; binding agent representations, propositional attitudes, and propositional content; and decoupling mental state representations from primary representations, among other processes) and executive function (EF), it is unclear what leads to the age-related deterioration of ToM ability. Does it come from the decline of ToM-specific competence, EF, or both? Here we explore the underlying causes of age-related decline in ToM ability.

EF is associated with the development of ToM across the lifespan. For example, aspects of EF including inhibitory control and working memory have been shown to be correlated with false-belief reasoning in early development [2]. It is also well established that EF deteriorates with age [3], and although this alone does not establish that it is responsible for changes in ToM among the elderly, it has been also found that the age-related decline of ToM is related to the deterioration of EF [4–8]. For example, EF is significantly correlated with performance on ToM tasks [6] and even statistically mediates age-related decline of ToM [4,5,7,8]. While suggestive, these results are unable to establish whether older adults’ difficulty in ToM is caused by reduced EF or by decline in ToM competence.

ToM ability in the elderly has been primarily assessed with elicited-response (or explicit) tasks [4–8]. In a common variant, participants observe an object moved in the presence or absence of an agent and then are prompted to provide a verbal response indicating where the agent thinks the object is located. Task analyses suggest these conditions impose high EF load (e.g., inhibiting a prepotent response, [9]).

One previous meta-analysis [10] reviewed existing studies on aging and ToM to see whether there is age-related decline of ToM in a variety of ToM tasks by categorizing tasks according to domain (i.e., cognitive, affective, or mixed ToM) and modality of presentation (i.e., dynamic-visual, static-visual, or verbal). The results showed that older adults performed more poorly than younger adults across all types of ToM tasks, and it was concluded that ToM decline with age is due to the elderly’s real deterioration of ToM competence, not due to performance factors. However, the meta-analysis does not answer whether changes in underlying ToM competence, EF, or both cause age-related decline of ToM. Critically, since the tasks entered into the meta-analysis were elicited-response ToM tasks, which impose high executive demands, it leaves the possibility open that the high performance demands of those tasks masked an intact, underlying mindreading capacity in the elderly.

In contrast to the elicited-response tasks, spontaneous-response (or implicit) tasks reduce response-selection and response-inhibition [11]. For example, spontaneous-response tasks (e.g., looking behavior, spontaneous helping behavior) do not require participants to explicitly generate their responses, needing less non-ToM processing compared to the elicited-response ToM tasks. During the tasks, participants’ spontaneous response (e.g., looking behavior) is measured. In the current study, we use a spontaneous-response ToM task to explore whether the elderly show improved ToM ability when performance demands are reduced.

An EF-decline account, in which decline in underlying EF is responsible for reduced ToM ability in the elderly, produces two logically connected predictions. First, individuals with compromised EF, such as the elderly, should have difficulty on ToM tasks that put high demands on EF. The first prediction is supported by many previous studies [4–8]. These studies all relied on explicit elicited-response tasks which impose high performance demands, including EF load. These increased demands appear to overwhelm the limited EF in the elderly, producing age-related deterioration in ToM ability. Second, individuals with compromised EF should show improved performance on ToM tasks that reduce load on EF. While these low demand tasks have been used extensively with infants and children [12–14], they have not to be widely used with older adults. The current investigation tests the second prediction. If the EF-decline account is correct, it is predicted that the elderly in the current study would show comparable performance to younger adults on a spontaneous-response task which involves low EF demand.

In contrast, a competence-decline account, in which decline in ToM competence is responsible for reduced ToM ability in the elderly, predicts worse performance on ToM tasks in the elderly compared to younger adults for both elicited-response and spontaneous-response tasks. Reduced demands should be of no benefit if the elderly lack the concepts and mechanisms to represent and compute mental states.

This sets up a critical test: If ToM competence in the elderly is intact as the EF-decline account claims, then reducing EF demands should improve ToM performance, but if ToM competence is not intact as the competence-decline account claims, then reducing EF demands should not improve ToM performance. To adjudicate between the accounts, we explored what happens in a low-demand task.

A recent study found that both older and younger adults performed similarly on a spontaneous-response ToM task, consistent with the EF-decline account that older adults have preserved false-belief tracking [15]. The current study differed in three critical respects: 1) the research was preregistered, supporting valid null hypothesis significance testing by controlling long-run error rates, 2) Bayesian analysis was used to quantify evidence for a null hypothesis of no difference in belief tracking between older and younger adults relative to an alternative hypothesis that there was a difference, and 3) EF differences between older and younger adults were measured, not inferred.

## Materials and methods

The Non-Medical Research Ethics Board at the University of Western Ontario approved the study (approval number 107251). Written consent was obtained. We preregistered the hypotheses described above, an a priori power analysis, and data analysis plans. Preregistration, stimuli, processed data, and data analyses are available on the Open Science Framework: [https://osf.io/y7xrq/?view_only=e0fe507f7d9d4420a98b2152d91c2481]. Because the raw data contain potentially identifying and sensitive participant information, they were not institutionally approved by the Non-Medical Research Ethics Board to be shared on a repository. Data requests can be sent to ethics@uwo.ca; data are also directly available upon request from either co-author.

### Participants

Sixty-seven younger adults and 68 older adults participated in the study. Thirty-seven younger adults were recruited through a university research participation pool and received course credit for their participation, and the rest were recruited via poster on campus and were paid for their participation. The older adults were recruited from the local community and were paid for their participation. Prior to collecting data, we conducted an a priori power analysis using G*Power 3.1 [16]. Based on an a priori power analysis with 80% power, a medium effect size, and an alpha value of .05, we needed 98 participants (49 participants per each age group).

Out of 135 participants, 37 (18 younger and 19 older adults) were excluded for further analysis due to failure to look at both object and empty locations in at least one condition (*n* = 12, 4 younger and 8 older adults), having a current diagnosis or history of major psychiatric and neurological illnesses (*n* = 13, 5 younger and 9 older adults), self-reporting as English as a second language (3 younger adults), failing to complete several measures (1 younger adult), or demonstrating signs of depression (*n* = 7, 5 younger and 2 older adults) measured on the short Geriatric Depression Scale (sGDS; [17]). For two younger adults, data was obtained from only the first half of the ToM task (see Procedure section), but their data was included as it met our pre-registered inclusion criteria. As a result, the final sample consisted of 49 (34 females) healthy younger adults (*M* = 20.37, *SD* = 3.25, 25 from the Participation Pool) and 49 (37 females) healthy older adults between 60 to 87 years of age (*M* = 69.37, *SD* = 7.58). Older adults reported more years of education than the younger adults (older: *M* = 16.03, *SD* = 3.24; younger: *M* = 14.08, *SD* = 2.48, *t*(96) = 3.34, *p* = .001). Also, older adults showed higher scores in verbal subtest of Wechsler Abbreviated Scale of Intelligence (WASI; [18]) compared to the younger adults (older: *M* = 41.69, *SD* = 7.65; younger: *M* = 37.47, *SD* = 6.48, *t*(96) = 2.95, *p* = .004). The two age groups were not significantly different in the Mini Mental State Examination (MMSE; [19]), *t*(59.815) = 1.886, *p* = .064. Regarding sGDS, older adults showed lower scores than the younger adults (older: *M* = 0.59, *SD* = 0.96; younger: *M* = 1.96, *SD* = 1.56, *t*(77.730) = 5.20, *p* < .001).

### Tasks and materials

#### General cognitive assessment tasks

To ensure that all participants had normal mental functioning, participants were asked to complete a simple questionnaire to obtain their demographic data, the MMSE for screening dementia, verbal subtest of the WASI for obtaining an estimate of their crystalized IQ, and the sGDS for screening any symptoms of depression.

#### Theory of mind (ToM) eye tracking task

Forty animations (20 true-belief and 20 false-belief trials) varying in child identity, animal identity, initial animal location, and room identity, were used. For half of the 40 animations, a child stood behind the box where s/he believed the animal was located at the end of a trial, whereas for the other half of the animations, the child stood behind the box where s/he did not believe the animal was located. Therefore, where the child stood was unrelated to his/her belief about the location of the animal.

There were four phases in each trial: Animation phase, Anticipation phase, Fixation phase, and Response phase. During the animation phase (11 seconds), a child (protagonist) put an animal in one of two boxes and left the room. At this time, on false-belief trials, the animal moved out of the box and into the other box during the child’s absence, whereas, on true-belief trials, the child returned the room first and then the animal moved out of the box and into the other box. When the child returned to the room on both types of trials, s/he walked forward, toward the boxes, but stayed in the middle. During the anticipation phase (4 seconds), the child stood still in the middle. During the fixation phase (1 second), a white fixation cross appeared on a grey screen. Lastly, during the response phase, the child stood behind one of the two boxes. At this time, participants were asked to press one of the buttons (left arrow key or right arrow key) to indicate whether the child stood behind either the left or right box.

To measure participants’ eye movements, a corneal reflection eye tracker (Tobii x120, Tobii Technology, Stockholm, Sweden) was used with E-prime software (version 2.0.8.90; Psychology Software Tools Inc., Pittsburgh, PA, USA) and E-prime Extensions for Tobii (version 2.0.1.26). Specifically, the amount of time participants looked at each of the two boxes during a five-second-analysis window (including the anticipation phase and the fixation phase), from after the child returned to the room to before she/he was about to approach one of the boxes, was measured and used to compute a preferential looking score ranging from 0 (looking only to the animal box) to 1 (looking only to the empty box). Since the child believed the animal was in the empty box on false-belief trials and in the animal box on true belief trials, if participants spontaneously track those beliefs and make anticipatory eye movements to the location where they expect the child to search given those same beliefs, they should look more to the empty box on false-belief trials, than on true-belief trials.

#### Executive function (EF) tasks

Since executive function (EF) refers to a set of multiple cognitive processes for goal-directed behavior [20, 21], using a variety of EF tasks is crucial to measure EF. One of the widely used models proposes that EF consists of three subcomponents including inhibition, updating, and shifting [21, 22]. Based on this model, we measured each of the three subcomponents of EF with two or more standard tasks in case one task did not fully measure a specific subcomponent. For inhibition, we used Stroop and Go/No-go tasks; for updating, we used forward and backward digit span tasks; for shifting, we used Digit Symbol Substitution Test (DSST, [23]), Trail Making Test (TMT, [24]), and Wisconsin Card Sorting Test (WCST, [25]). In the Stroop task, participants were asked to respond to color of a word, not to the name of the word, as quickly and accurately as possible. There were 180 trials (60 congruent trials, 60 incongruent trials, and 60 neutral trials). The reaction time and accuracy for each trial were recorded. In the Go/No-go task, participants were asked to press the Enter key when the Go condition was met (i.e., whenever the letter ‘W’ appears on a monitor) and not to press anything when the Go condition was not met (No-Go condition). There were 300 trials in total, and 45 trials out of them were the No-Go condition. Accuracy was recorded, and the false-alarm rate was analyzed. In the DSST, participants were asked to attend to the symbol associated with each of nine different digits and then to draw the corresponding symbols under each digit as quickly as possible in 90 seconds. The number of correct answers was recorded. Also, in the Digit Span tasks (forward and backward) measuring working memory, participants heard a series of numbers and they were asked to recall the numbers in sequence (or in reverse of the presented order). There were three trials in each section (e.g., in section 2, two numbers were presented, and three numbers were presented in section 3). If they correctly recalled one of the three trials within the same section, the task went on. If they gave wrong numbers on all the trials within the same section, the task was stopped. The number of correct answers was analyzed. In the TMT, there were two parts to the test: in Part A, participants were required to connect letters in alphabetical order as quickly as possible and in Part B, they had to connect letters and numbers alternatingly in alphabetical (letters) and ascending (numbers) order, respectively (e.g., A1B2C3). Since Part B has been considered an EF measurement, the total time to complete Part B was measured. Lastly, for the WCST, the Psychology Experiment Building Language tests (PEBL)’s version of the WSCT [25] was used. Accuracy was analyzed.

### Procedure

The study proceeded in the following order: General cognitive assessment tasks, calibration phase for the eye tracking task, ToM eye tracking task (the first session; 20 trials), half of the EF tasks, 10-minute break, ToM eye tracking task (the second session; 20 trials), a questionnaire to ensure whether they understood the eye tracking task’s instructions, and the remaining half of the EF tasks. Half of the participants were given the Stroop task, Trial making task, and Digit span task as the first half of the EF tasks, whereas the other half of the participants were given Go/No-go task, Digit symbol substitution task, and Wisconsin card sorting task as the first half of the EF tasks.

## Results

**Fig 1:**
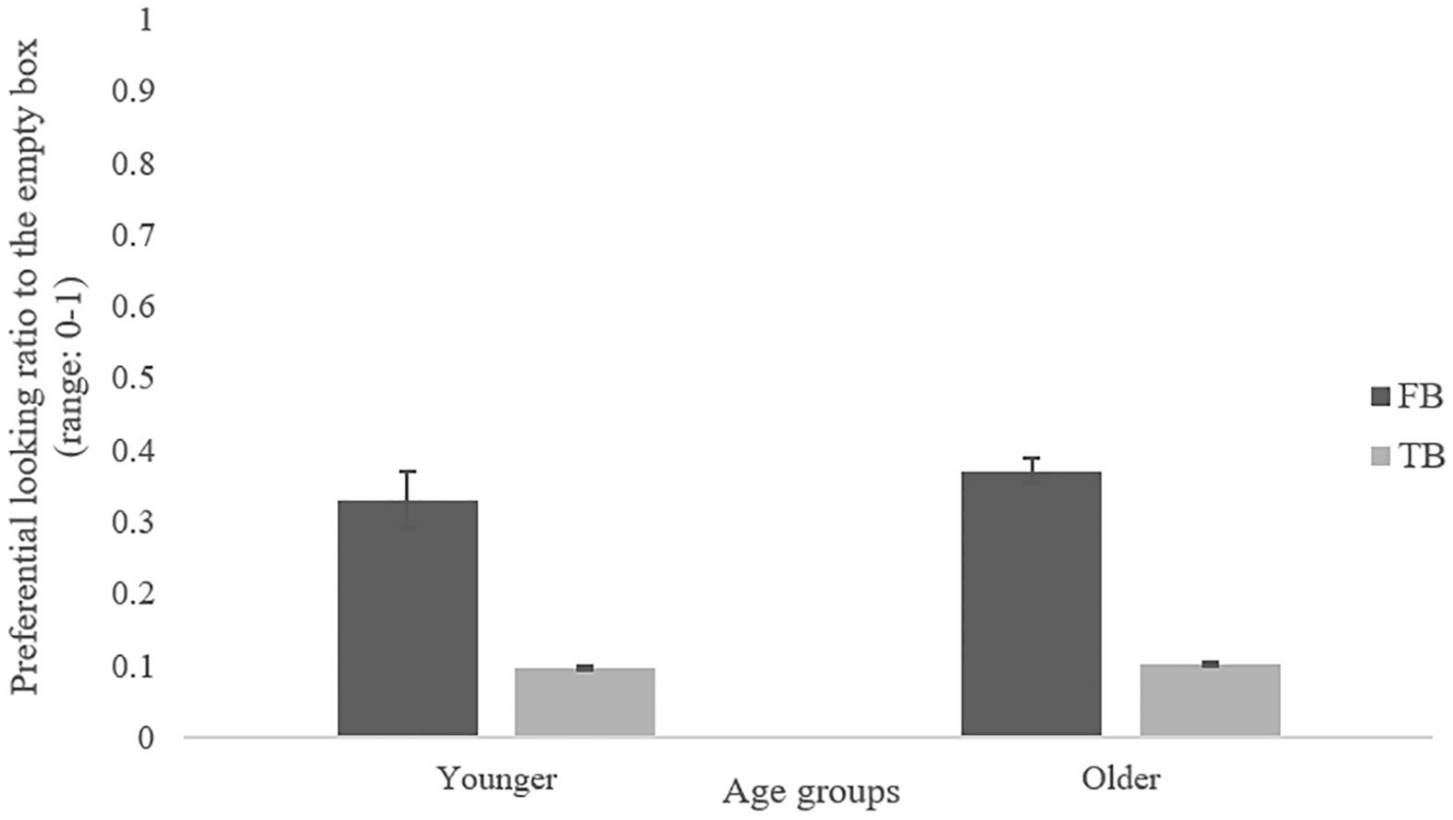
Preferential looking ratios, with a score of 0 meaning looking only to the animal box, a score of 1 meaning looking only to the empty box. Error bars represent standard errors.

For the ToM eye tracking task, the ratio of the participants’ looking time to the empty box on the false-belief trials (or the true-belief trials) to the total looking time to both the empty and object box on the false-belief trials (or the true-belief trials) was calculated, producing a looking preference score to the empty box for both conditions (Fig 1).

### Age differences in spontaneous belief-reasoning

As a confirmatory analysis, age effects on preferential looking ratio to the empty box were investigated using a 2 (age group: younger vs. older adults) X 2 (belief type: true-belief vs. false-belief) mixed design ANOVA. There was a significant main effect of belief type, *F*(1, 96) = 63.61, *p* < .001, $\eta_{p}^{2}=.399$, showing that looking preference to the empty box was larger in false-belief trials (*M* = 0.35, *SD* = 0.29) than in true-belief trials (*M* = 0.10, *SD* = 0.14). This indicates that participants processed the child’s belief about the location of the animal. No main effect of age group, *F*(1, 96) = 0.47, *p* = .493, and no interaction effect between age group and belief type, *F*(1, 96) = 0.28, *p* = .598, were found. Further, the Bayes Factor (with a default Cauchy prior width of 0.707, [26]) suggested positive evidence for null hypotheses about the age effect, BF_01_ = 4.999, and the interaction effect, BF_01_ = 3.95 relative to the alternative hypotheses of age effect and interaction effect, respectively.

### Age differences in the EF tasks

As a confirmatory analysis, descriptive information on each age group’s performance on each EF task (raw scores) and the statistical differences for each comparison is shown in Table 1. For the analysis, dependent measures from all the EF tasks were standardized. The younger adults significantly outperformed the older adults on all the EF tasks except the Go-Nogo task and the Digit Span task (both forward and backward).

**Table 1:** Descriptive information and t-test analyses of age-related differences on EF tasks.

	Younger		Older		Group difference			
	M	SD	M	SD	*t*	*df*	*P*	*Cohen’s d*
*Inhibition*								
Stroop effect^a^ (s)	0.10	0.10	0.22	0.19	-3.64	66.813	.001	-0.89
Go/No-go^b^ (%)	4.14	2.38	3.53	2.32	1.27	94	.206	0.26
*Updating*								
Digit Span: Forward^c^	18.80	2.90	18.60	3.72	0.28	95	.777	0.06
Digit Span: Backward^c^	12.10	3.20	11.15	3.57	1.39	95	.168	0.29
*Shifting*								
Digit Symbol Substitution^c^	50.76	12.14	34.25	7.65	7.88	75.004	< .001	1.82
TMT_B^d^ (s)	57.22	21.35	79.09	31.55	-4.02	96	< .001	-0.82
WCST^c^ (%)	76.40	11.48	69.92	12.94	2.60	94	.011	0.54

^a^ median reaction time (RT) for neutral condition—the median RT for incongruent condition

^b^ false alarm rate

^c^ the number of correct answers (cf. Digit Symbol Substitution: within 90 seconds)

^d^ the total time to complete the part

### Spontaneous belief-reasoning and EF

As an exploratory analysis, correlation analyses between EF and the looking scores in the false belief condition were explored (we thank a reviewer for this recommendation). There was no significant correlation between the looking scores in the false belief condition and EF (all *p*s > .10). In addition, the Bayes Factor suggested positive evidence for null hypotheses concerning the correlation relative to the alternative: Go-Nogo (BF_01_ = 6.231), Stroop (BF_01_ = 4.740), the updating composite score (BF_01_ = 3.593), and the shifting composite score (BF_01_ = 7.583), suggesting no significant association between EF measurements and false-belief reasoning.

When correlations between the looking scores in the false belief condition and EF were broken down by age group, in the older group, there was no significant correlation between the looking scores in the false belief condition and EF (*p*s>.090) except for the updating composite score (r = 0.30, p = .041). In the younger age group, there was no significant correlation between the looking scores in the false belief condition and EF (all *p*s >.50).

However, this analysis should be interpreted with caution for several reasons. First, ToM and EF could still be related if other unmeasured variables weaken the relationship or even pull the relationship in the other direction, washing out the correlation. Second, while a weak or lack of a correlation is consistent with reduced executive demands in the spontaneous-response task, a strong correlation would still be consistent with lowered executive demands because the claim is not that spontaneous-response tasks eliminate executive demands, just that they sufficiently reduce them to reveal underlying competence better than their elicited-response task counterparts.

## Discussion

The current study examined whether older adults, known to show age-related decline in ToM ability when given tasks that impose high performance demands [4–8], would track belief when using a spontaneous-response task that reduced non-ToM processing. The present finding suggests that older adults track belief when EF demands are sufficiently lowered, revealing intact underlying ToM competence in the elderly. Although recent work appears to show a similar result [15], lack of preregistration and use of p > .05 to argue for no differences in belief tracking between younger and older adults limit the validity of those results. The current study was preregistered and used Bayesian model comparison to support valid inference.

This work has several implications. It addresses competence and performance factors in ToM processing and aging. The elderly’s intact mentalizing ability in the current study implies that the age-related deterioration of ToM that the previous studies have shown comes from the elderly’s difficulty in expressing their ToM competence, not from decline of ToM competence. This study suggests that EF seems to be the underlying sources of age-related deterioration of ToM that the previous literature have shown.

Several limitations should be considered. First, the older adults in this study had relatively higher years of education and WASI scores compared to the younger counterparts, so it is possible that their relatively high functioning allowed them to compensate for an otherwise reduced ToM capacity in the spontaneous-response ToM task (e.g., by using non-mentalistic strategy to predict others’ behavior). Closer matching of participants in future research would address this concern. Second, even though numerous studies have shown age-related decline of ToM with various elicited-response ToM tasks, directly comparing older adults’ performance on elicited-response ToM tasks and closely matched spontaneous-response ToM tasks would be necessary to strongly claim the existing ToM tasks have heavy EF demands for future studies (cf. [15]). Third, not all agree on how to interpret performance in spontaneous-response tasks. This study assumes they tap a conceptual ToM system rather than a “belief-like” or non-mentalistic capacity [27, 28], and although recent work casts doubt on these alternative accounts (e.g., [29, 30]), this assumption is still being debated and tested. Lastly, although the current study suggests that some aspects of core ToM mechanisms are intact in the elderly, ToM involves multiple sub-systems each with multiple processes and concepts [27, 31–34] and it is possible that concepts, processes, or sub-systems not engaged by the specific task used here are in fact compromised in the elderly.

This study helps illuminate age-related development of ToM, providing evidence that the deterioration of ToM ability with age is caused by changes in EF, not in ToM competence. While the competence-performance distinction has long played a role in explaining the acquisition of abilities in early development (e.g., [35]), the current work shows the distinction can be put to work to explain changes in later development as well.
